# Neoadjuvant therapy with eribulin, doxorubicin and cyclophosphamide for patients with HER2-negative inflammatory breast cancer: a phase II study

**DOI:** 10.1186/s13058-025-02108-4

**Published:** 2025-09-29

**Authors:** Kristina Fanucci, Eren D. Yeh, Ruichao Shi, Lei Qin, Camden P. Bay, Molly DiLullo, Ashka Patel, McKenna Moore, Zachary T. Herbert, Beth T. Harrison, Faina Nakhlis, Jennifer Bellon, Laura Warren, Jennifer L. Guerriero, Sara M. Tolaney, Meredith Regan, Beth Overmoyer, Steven Van Laere, Filipa Lynce

**Affiliations:** 1 Department of Medical Oncology, Dana-Farber Cancer Institute, 450 Brookline Avenue, Boston, MA USA; 2 Harvard Medical School, Boston, MA USA; 3 Brigham and Women’s Hospital, Boston, MA USA; 4 Takeda Pharmaceuticals, Cambridge, MA USA; 5 Center for Oncological Research (CORE) and Integrated Personalized & Precision Oncology Network (IPPON), University of Antwerp, Antwerp, Belgium

**Keywords:** Eribulin, Doxorubicin, Cyclophosphamide, Neoadjuvant therapy, HER2-negative, Inflammatory Breast Cancer

## Abstract

**Background:**

Inflammatory breast cancer (IBC) is an aggressive and highly angiogenic disease. Eribulin is a microtubule inhibitor with anti-angiogenic properties.

**Methods:**

In a phase II trial, we examined the efficacy of an eribulin-containing neoadjuvant regimen (eribulin- > doxorubicin plus cyclophosphamide (AC) or AC- > eribulin) for patients with newly diagnosed HER2-negative IBC. Pathologic complete response (pCR: ypT0/Tis ypN0) was the primary endpoint; residual cancer burden (RCB) categories were also recorded. Five patients from each cohort underwent dynamic contrast enhanced MRI (DCE-MRI) and diffusion weighted MRI. All patients had research breast biopsies for transcriptomic, differential gene expression, and cell subset analysis at baseline and one week after the first dose of therapy.

**Results:**

19/22 (86.4%) patients had hormone receptor-positive disease. All patients were able to undergo planned curative-intent surgery and radiation. One patient had a pCR, and long-term outcomes were encouraging: after median follow up of 76 months, 3 patients experienced disease recurrence. Five-year event-free survival (EFS) was 85.6%. The regimen was tolerated with expected side effects—the most common grade 1 or 2 AEs were fatigue (95.5%), nausea (68.2%), and alopecia (63.6%). Seven out of 22 (31.8%) patients experienced any grade 3 or 4 AE, with neutropenia (22.7%) being the most common. DCE-MRI showed decreased tumor vascularization after 1 week of treatment versus baseline. Transcriptomic analysis using quantification of synthesized dsDNA libraries and tumor microenvironment analysis of paired baseline and on-treatment samples showed residual cancer burden (RCB)-III tumors were more likely to have genes associated with adipogenesis/fatty acid metabolism and cells associated with immunosuppression.

**Conclusions:**

Despite the low pCR rate, all patients were able to undergo curative surgery, and long-term outcomes were encouraging with 5-year EFS 85.6%. Decreases in tumor vascularization with treatment were detected by DCE-MRI parameters irrespective of initial chemotherapy received. Adipogenesis/fatty acid metabolism and cells associated with immunosuppression are potential mechanisms of resistance and targets for future investigation in this unique patient population.

**Trial registration:**

ClinicalTrials.gov (NCT02623972)(Registration date: 12/02/15).

**Supplementary Information:**

The online version contains supplementary material available at 10.1186/s13058-025-02108-4.

## Introduction

Inflammatory breast cancer (IBC) is a rare presentation of breast cancer with a poor prognosis that contributes significantly to breast cancer-related mortality [1]. Although IBC remains understudied, in part because it accounts for just 2–5% of all invasive breast cancers [2], it is highly angiogenic and displays mesenchymal features that promote rapid metastasis [3]. Owing to the skin involvement of IBC, it is not considered resectable at initial presentation. Therefore, neoadjuvant chemotherapy has become the standard treatment for IBC [2]. Most neoadjuvant systemic therapy (NAST) regimens in IBC include anthracyclines and taxanes. However, an optimal NAST for HER2-negative IBC has yet to be determined [4–6].

Eribulin mesylate is a synthetic derivative of the natural product halichondrin B that exerts anti-cancer activity through a microtubule-destabilizing anti-mitotic mechanism of action [7, 8]. Preclinical data suggest that eribulin induces two specific biological effects in breast cancer: reversion of the epithelial-mesenchymal transition (EMT) phenotype by reducing expression of EMT-associated genes and normalization of the tumor vasculature by increasing tumor perfusion and reducing expression of angiogenesis-related genes [9]. The open-label phase III EMBRACE trial compared eribulin therapy with treatment of physician’s choice (TPC) among patients with heavily pre-treated, locally advanced unresectable or metastatic breast cancer. Patients who received eribulin had a significant improvement in overall survival (OS) compared with patients who received TPC [10].

Guided by the above findings, we hypothesized that the use of eribulin would result in a meaningful improvement in the pathologic complete response (pCR) rate in patients with HER2-negative IBC, a disease that exhibits some degree of resistance to chemotherapy [5]. We therefore conducted a phase II study to evaluate the efficacy, assessed by pCR rate, of an eribulin-containing regimen for IBC. We also assessed toxicity and long-term survival outcomes. Based on pre-clinical data revealing changes in dynamic contrast-enhanced magnetic resonance imaging (DCE-MRI) parameters after exposure to eribulin thought to reflect tumor vasculature remodeling [9] we also conducted an imaging sub-study with DCE-MRI.

## Methods

### Study design and patient population

This prospective phase II clinical trial examined NAST containing eribulin (E), and doxorubicin and cyclophosphamide (AC) for patients with newly diagnosed HER2-negative IBC. Patients were eligible for inclusion in the study if they were ≥ 18 years of age and diagnosed with IBC with no evidence of metastatic disease in the viscera or bone (T4d, stage III); distant nodal involvement only was allowed (Stage IV). HER2 status was assessed by immunohistochemistry (IHC) and/or in situ hybridization (ISH) according to the 2013 American Society of Clinical Oncology/College of American Pathologists (ASCO/CAP) guidelines [11]. Patients were excluded if they were receiving any other investigational drug, were pregnant, had a history of an allergic reaction to similar drugs, had an ongoing or active infection, were human immunodeficiency virus (HIV)-positive, or had a history of another malignancy.

### Compliance with ethical standards

This study was conducted in accordance with the International Conference on Harmonization Good Clinical Practice Standards and the Declaration of Helsinki. Institutional review board approval was obtained at Dana-Farber/Harvard Cancer Center (DF/HCC). Monitoring of trial progress and safety was reviewed twice yearly by the DF/HCC independent data safety monitoring committee (DSMC). All patients provided written informed consent prior to initiating any study treatments or procedures. This trial was registered with ClinicalTrials.gov (NCT02623972).

### Treatment plan

Patients in Cohort A received E 1.4 mg/m^2^ given on day 1 (D1) and day 8 (D8) for four cycles of 21 days. This was followed by dose-dense doxorubicin (A) 60 mg/m^2^ and cyclophosphamide (C) 600 mg/m^2^ given every two weeks for four cycles. Patients in Cohort B received a reverse of cohort A with four cycles of AC followed by 4 cycles of E.

Following completion of NAST, patients who were deemed operable underwent modified radical mastectomy (MRM) and postmastectomy radiation therapy (PMRT). Adjuvant endocrine therapy (ET) was administered per standard of care. Patients were followed for disease recurrence/progression and survival.

### Clinical evaluation

Patients received baseline imaging with either mammogram/ultrasound or clinical breast MRI per standard practice evaluations. Patients were evaluated with physical exam each cycle during pre-operative therapy and clinical response was assessed based on physical exam. Patient response was characterized as progressive disease, stable disease, partial response, or complete response. Repeat mammogram/ultrasound or clinical breast MRI was completed prior to surgery. Breast imaging and clinical exam were used to determine surgical resectability.

### Statistical considerations and analysis

The primary objective was to estimate the pCR rate, defined as no invasive disease in breast and axillary lymph nodes, irrespective of ductal carcinoma in situ (ypT0/Tis, ypN0), following NAST with E followed by AC. Residual cancer burden (RCB) categories after preoperative therapy were determined, as defined by Symmans et al*.* [12]. Secondary endpoints included toxicity assessment of protocol therapy, and overall survival (OS) defined as time from surgery to death from any cause.

Patients were enrolled in cohort A according to a Simon’s minimax two-stage design (α = 0.10, β = 0.10), requiring at least 2 of 16 patients to experience pCR to proceed to complete enrollment of 25 patients, to reject a null hypothesis pCR rate of ≤ 10% in favor of a pCR rate ≥ 30% [6]. More recent literature and the DFCI experience [13] suggest lower pCR rates in estrogen receptor-positive (ER +)/HER2-negative than ER-negative/HER2-negative breast cancer (TNBC), which were the majority in cohort A. Hence cohort B was planned to reject a null hypothesis pCR rate of ≤ 2% in favor of pCR rate of ≥ 23%, requiring at least 1 of 12 patients to have pCR to proceed to complete enrollment of 16 patients (α = 0.10, β = 0.10). The pCR and RCB rates were reported with two-sided exact binomial 80% confidence interval (CI).

Adverse event (AE) monitoring and reporting in this trial included cycle, pre-surgery, and post-surgery visit reports. The descriptions and grading scales found in the revised NCI Common Terminology Criteria for Adverse Events (CTCAE) version 4.0 were utilized for AE reporting.

### Sample collection

All patients were required to undergo two research tumor biopsies of the affected breast for correlative studies on day 1 (D1) (pretreatment) and day 8 (D8), using a 14-gauge core needle to obtain 4–6 core specimens under ultrasound guidance. A sample of any residual disease within the breast at the time of surgery was obtained. Fresh tumor tissue was immediately embedded in optimal cutting temperature (OCT) medium for frozen sections of tissue and stored at −80 °C to ensure maintenance of tissue integrity.

### Transcriptomic analysis

RNA was extracted from tumor biopsy specimens with > 50% tumor cellularity. Complementary DNA (cDNA) was generated using reverse transcription enzyme (RT) as per standard protocols. The expression of 10 EMT-related genes (*TGFB1, TGFB2, CDH1, KRT18, CDH2, VIM, TWIST1, SNAI2, ZEB1, ZEB2*) and 15 angiogenesis-related genes (*VEGFA, VEGFR1, VEGFR2, VEGFR3, PGF, DLL4, JAG1, NOTCH4, EFNB2, EPHA2, EPHB1, WNT5A, WNT11, BPM4, CA9)* was determined in each sample by RT-quantitative polymerase chain reaction (RT-qPCR). All RT-qPCR raw data were stored in an electronic database within the Department of Cancer Biology, Dana-Farber Cancer Institute.

RNA was stored at −80 °C until downstream transcriptomic analysis. cDNA was synthesized using Takara SmartSeq v4 reagents from 2 ng of RNA (Takara Bio USA, Inc, San Jose, CA, USA). Full length cDNA was fragmented to a mean size of 200 bp with a Covaris R230 ultrasonicator and Illumina sequencing Libraries were prepared from 2 ng of sheared cDNA using IDT xGen DNA library prep reagents (Integrated DNA Technologies, Inc., Coralville, IA, USA) on a Beckman Coulter Biomek i7 according to manufacturer’s protocol (Beckman Coulter Life Sciences, Indianapolis, IN, USA). The finished dsDNA libraries were quantified by Qubit fluorometer (Thermo Fisher Scientific, Waltham, MA, USA) and Agilent TapeStation 2200 (Agilent Technologies, Inc., Santa Clara, CA, USA). Uniquely dual indexed libraries were pooled in equimolar ratios and evaluated for cluster efficiency and pool balance with shallow sequencing on an Illumina MiSeq (Illumina Inc., San Diego, CA, USA). Final sequencing was performed on an Illumina NovaSeq 6000 (Illumina Inc., San Diego, CA, USA) with paired-end 150 bp reads at the Dana-Farber Cancer Institute Molecular Biology Core Facilities. HISAT2 was used to map reads to the human reference genome (version Hg38) and samtools was used to convert resulting SAM files to sorted BAM files. RSEQC was used for quality control of the read mapping. The summariseOverlaps-function from the BioC-package GenomicAlignments was used to count the number of mapped reads per genomic location in the intersection non-empty mode taking unpaired reads and strand information into account. Finally, further analysis was conducted after all genes with raw expression counts above 10 in at least 10% of the cases (N = 18,179) were filtered in.

### Differential gene expression analysis

For the purpose of gene expression analysis and given that the only patient who experienced a pCR did not have tumor tissue from biopsies available for analysis, we grouped patients into those who had RCB I or II at surgery (RCB-I/II), and those who had RCB-III.

In order to identify differentially expressed genes among these groups, raw count data per sample were first scaled to their respective library sizes using the Trimmed Mean of M-values (TMM) method (BioC-package edgeR). Then, the mean–variance relationship was estimated using the voom-method (BioC-package limma) incorporating a design matrix with the contrast between RCB-I/II and RCB-III as main effect and a term modelling the interaction between these groups and measurement timepoint (i.e., pre-treatment baseline (day 1) and day 8 on-treatment). In addition, the estimated mean–variance trend was adjusted for repeated measures by providing a genome-wide consensus correlation coefficient estimated using the duplicateCorrelation function (BioC-package limma). Gene-wise weights estimated by modelling the mean–variance relationship were used in the linear modelling process to adjust for heteroscedasticity. Results are shown in volcano plot format.

To translate differences in gene expression into biological concepts, gene set enrichment analysis (GSEA) was performed for the hallmark gene set (Molecular Signatures Database – Broad Institute). Genome-wide vectors of log2-transformed gene expression changes between conditions of interest were analyzed using the fgsea-algorithm (BioC-package fgsea). Results are shown in volcano plot format modified for GSEA. For all analyses, nominal p-values < 0.05 were considered statistically significant.

### Cell subset enumeration

To explore differences in cellular composition between RCB-I/II and RCB-III at baseline and during treatment, gene expression data were deconvoluted using the R-package consensusTME in standard settings. ConsensusTME returns enrichment scores for 18 different cell types and automatically calculates a general immune score. For this analysis, a variance stabilizing transformation (VST) was applied to the raw count data using the VST function from the BioC-package DESeq2 using standard settings. Differences in enrichment scores for different cell types were calculated using generalized linear models (i.e., RCB-I/II vs. RCB-III either at baseline or day 8 on-treatment) or linear mixed effects models (i.e., baseline vs. on-treatment samples in either RCB-I/II or RCB-III) where appropriate using R-packages limma and lmerTest, respectively. Nominal p-values < 0.05 were considered statistically significant.

### RNA-seq mutation calling

To detect somatic mutations, HISAT2-aligned BAM files were used, and non-reference alleles were identified with freebayes. For this analysis, only the 93 genes reported in the catalogue of breast cancer genes [14] were considered, and their genomic coordinates were retrieved from Ensembl. Variant normalization was performed with bcftools and resulting VCF files were annotated using SnpEff. Finally, SnpSift was used to classify genomic variants according to dbSNP, ClinVar, and COSMIC. To identify relevant variants, annotated VCF files were filtered using the following criteria: only missense, Frameshift and stop-gain mutations reported in COSMIC were considered that were not reported as common variants in the 1000 genomes project and that were not classified as benign or Likely benign according to ClinVar or dbSNP. Only variants showing more than 17 variant allele reads were retained for further analysis. The filter on the number of variant alleles was determined by analyzing all mutations in our RNA-sequencing data set without records in dbSNP or ClinVar. In this set of mutations, sequencing errors will arguably be more prevalent. The average of variant reads in this call set was 4 with a standard deviation of 6.41. From these data, a cut-off on the minimal number of variant reads was calculated (i.e., 4 + 1.96 * 6.41 = 17), allowing to filter out 95% of these putative false positive variants. Results are shown in Oncoprint format generated with the R-package ggplot2. The number of mutations per sample was compared between baseline and on-treatment samples in RCB-I/II and RCB-III separately.

### Imaging analysis

Five patients from Cohort A and five patients from Cohort B who consented to undergo baseline and follow up MRI participated in an imaging research sub-study. MRI was performed at baseline and prior to C1D8. The timing of the second MRI was determined based on preclinical data suggesting early vascular remodeling with E detected by MRI [9].

MRI was performed on a 3 T System using an 8-channel breast coil (Prisma, Siemens Healthcare, Germany). Pre-contrast T1 mapping 3D fast spoiled gradient recalled echo (FSPGR) sequences were acquired using the variable flip angle method [15] with TR = 4.3 ms, TE = 1.8 ms, matrix = 256 × 160, slice thickness = 5 mm, and flip angles = 2˚, 4˚, 6˚, 10˚, 15˚. DCE-MRI imaging was obtained using the same FSPGR sequence with identical TR, TE, imaging matrix, slice thickness and flip angle = 8˚. Temporal resolution was 9.4 s and a total of 60 frames were acquired. A standard bolus (0.1 mmol/kg) contrast agent gadobutrol (Gadavist, Bayer HealthCare, Wayne, NJ) was injected intravenously with a power injector at a rate of 3 mL/sec 40 s after the DCE sequence was started. The bolus was followed by a saline flush of 20 mL injected at the same rate. T1 map was calculated from the variable flip angle gradient sequences. Signal intensity-time curve was converted to contrast agent (CA) concentration–time curve based on the measured T1 relaxation times and the longitudinal CA relaxation coefficient [16]. The dynamic concentration–time curve was fitted with a two-compartment pharmacokinetic (PK) model to generate the volume transfer constant (K^trans^) from blood plasma to extravascular extracellular space (EES), the fractional volume of EES (V_e_), and the rate constant between EES and blood plasma (K_ep_) [17]. A population-based arterial input function (AIF) was used in the PK model[18]. Area under the curve (AUC) from the signal intensity-time curve to 90 s after contrast enhancement [16] was also calculated as a nonparametric perfusion measurement. The analysis was performed using a research package MIStar (Apollo Inc. Australia) [15–18]. Diffusion weighted imaging (DWI) was acquired with a single-shot EPI sequence. TR = 6800 ms, TE = 81 ms, field of view (FOV) = 33*27 cm, matrix = 128*83. Spectral Adiabatic Inversion Recovery (SPAIR) was used for fat suppression, two b values were used, 50 s/mm^2^ and 850 s/mm^2^, each with 8 number of averages to achieve good signal. ADC map was calculated automatically from the scanner using mono-exponential fitting.

Research biopsies were taken prior to the start of imaging. A pre-treatment (baseline) breast MRI was acquired, followed by a second scan approximately 6–8 days after receiving the first therapeutic dose (on-treatment) of E (Cohort A) or dose-dense doxorubicin plus cyclophosphamide (Cohort B). Data were analyzed by comparing tumoral changes from baseline to on-treatment images using the following quantitative parameters: K^trans^, K_ep_, the incremental area under the incremental area under the signal time intensity curve (iAUC) and ADC. The change in these quantitative measurements between baseline and on-treatment was evaluated using an exact two-sided Wilcoxon signed-rank test. The magnitude of the changes was also reported according to cohort but not compared because of small sample size.

## Results

### Enrollment and clinical characteristics of participants

A total of 16 patients were enrolled in the study from February 26, 2016, to March 8, 2018; these patients constituted Cohort A. An additional 6 patients were enrolled from April 10, 2019, to December 2, 2020; these patients constituted Cohort B. Because only 1 patient in Cohort A achieved a pCR, enrollment of additional patients into Cohort A ceased. Enrollment in Cohort B closed after 19 months due to slow accrual.

Participant demographics and clinical characteristics are shown in Table 1 and Supplementary Table 1 (see Additional File 1). The median age of all participants was 58 years, and most (n = 19, 86.3%) had ER +, ≥ 1% immunostaining disease. All but one patient had stage III disease; one patient in Cohort B had de novo stage IV disease (nodal disease only).

**Table 1:** Patient and disease characteristics

		Treatment Cohort				Overall	
		Eribulin>ddAC		ddAC>Eribulin			
			%		%		%
N enrolled		16	100.0	6	100.0	22	100.0
Sex							
Female		16	100.0	6	100.0	22	100.0
Race							
White		16	100.0	5	83.3	21	95.5
Other		.	.	1	16.7	1	4.5
Ethnicity							
Non-Hispanic		16	100.0	6	100.0	22	100.0
BRCA mutation?							
No Mutation		13	81.3	5	83.3	18	81.8
No BRCA tested		3	18.8	1	16.7	4	18.2
Menopausal Status							
Premenopausal		6	37.5	2	33.3	8	36.4
Perimenopausal		1	6.3	.	.	1	4.5
Postmenopausal		9	56.3	4	66.7	13	59.1
ECOG Performance Score							
0		16	100.0	6	100.0	22	100.0
Histology							
IDC		13	81.3	5	83.3	18	81.8
IDC/ILC		2	12.5	1	16.7	3	13.6
Apocrine		1	6.3	.	.	1	4.5
Histologic grade							
1		1	6.3	1	16.7	2	9.1
2		6	37.5	2	33.3	8	36.4
3		9	56.3	3	50.0	12	54.5
LVI on biopsy?							
No		13	81.3	6	100.0	19	86.4
Yes		3	18.8	.	.	3	13.6
Multifocal tumor							
No		14	87.5	2	33.3	16	72.7
Yes		2	12.5	4	66.7	6	27.3
ER status	PgR status						
<1%	<1%	3	18.8	.	.	3	13.6
1-10%	<1%	.	.	1	16.7	1	4.5
>10%	<1%	2	12.5	.	.	2	9.1
	1-10%	2	12.5	.	.	2	9.1
	>10%	9	56.3	5	83.3	14	63.6
Clinical N stage							
N0		3	18.8	1	16.7	4	18.2
N1		10	62.5	4	66.7	14	63.6
N2		1	6.3	1	16.7	2	9.1
N3		2	12.5	.	.	2	9.1
Clinical M stage							
M0		15	93.8	5	83.3	20	90.9
M1		1	6.3	1	16.7	2	9.1
Clinical Stage							
IIIB		14	87.5	4	66.7	18	81.8
IIIC		2	12.5	1	16.7	3	13.6
IV		.	.	1	16.7	1	4.5

*Abbreviations: ddAC* dose-dense doxorubicin (Adriamycin) and cyclophosphamide (Cytoxan), *ECOG* Eastern Cooperative Oncology Group, *IDC* invasive ductal carcinoma, *ILC* invasive lobular carcinoma, *LVI* lymphovascular invasion, *ER* estrogen receptor, *PgR* progesterone receptor

### Clinical Outcomes

Nineteen patients completed the full course of NAST. Due to toxicity, one patient received four cycles of E followed by three cycles of dose-dense AC only, and two patients received three cycles of E prior to four cycles of AC. All 22 patients underwent MRM at 21 to 26 weeks after registration (median 3.6 weeks after completing NAST; range 2.3–7.1 weeks) and all 22 patients underwent PMRT. Nineteen patients with ER + disease received adjuvant ET in accordance with treatment guidelines.

None of the patients demonstrated progression of disease during NAST. Only one of the 16 patients in Cohort A had a pCR (6.25%, 80% CI: 0.70–22.22 Cohort A; 4.55%, 80% CI: 0.48–16.56 of the entire study population). Two patients had no invasive disease in the breast but had residual lymph node involvement (residual cancer burden (RCB)-I, RCB-II) (Table 2).

**Table 2:** Pathological response

		**Eribulin->ddAC** ***n=16***	**ddAC->Eribulin** ***n*=6**
**Pathologic complete response**			
	yes	1	0
	no	15	6
**Invasive disease in breast at time of surgery**			
	yes	13	6
	no	3	0
**Size of residual invasive disease in breast**			
	none	3	0
	0.1-0.5 cm	2	1
	0.6-1 cm	1	1
	1.1-2 cm	2	1
	2.1-5 cm	5	0
	≥5 cm	2	2
	unknown	1	1
**Number of positive axillary nodes**			
	0	3	0
	1-3	5	4
	4-9	7	1
	≥10	1	1
**Residual cancer burden (RCB)**			
	0	1	0
	I	2	0
	II	4	3
	III	9	2
	unknown	0	1

*Abbreviations: ddAC* dose dense doxorubicin (Adriamycin) and cyclophosphamide (Cytoxan)

Supplementary Table 2 in Additional File 1 shows the degree of clinical response by exam post-NAST and pre-surgery response by magnetic resonance imaging (MRI). Clinical response assessed by exam found complete response (CR) in 45.5% of all patients (n = 10), partial response (PR) in 50% of all patients (n = 11), and stable disease (SD) in one patient (4.5%). When the clinical response by exam was compared with MRI images pre-surgery, imaging results were in close agreement for patients with a CR (90% had PR) and PR (91% had PR); two patients identified as CR and PR by exam were identified as having SD by MRI.

The median follow-up from time of registration was 76 months (range 36.1–88.1 months). None of the patients had loco-regional recurrence. Distant recurrence/progression was reported in three patients. One patient (ER +, RCB-III) had bone metastasis 48 months after registration and 42 months after surgery. This patient was still alive 78 months after registration. The second patient (ER-, RCB-II) developed metastasis in the Liver and bone 22 months after registration and 17 months after surgery, with subsequent development of central nervous system metastasis. This patient died 37 months after registration. The third patient (stage IV at baseline, ER +, RCB-III) had disease progression with new lung and bone metastases 16 months after registration and 11 months after surgery and died 30 months after registration. Five-year event-free survival (EFS) was 85.6% (95% CI: 61.4%, 95.1%).

### Adverse events (AE)

The AEs are listed according to grade in Table 3. The most common grade 1 or 2 AEs were fatigue, nausea, and alopecia. Seven out of 22 patients experienced any grade 3 or 4 AE, with neutropenia being the most common.

**Table 3:** Reported adverse events

CTCAE v4.0		Max Grade			
		1	2	3	4
		N	N	N	N
Blood and lymphatic system disorders	Anemia	.	1	1	.
	Lymph node pain	.	1	.	.
Cardiac disorders	Palpitations	1	.	.	.
Ear and labyrinth disorders	Hearing impaired	1	.	.	.
	Tinnitus	1	.	.	.
	Vertigo	1	.	.	.
Eye disorders	Dry eye	.	1	.	.
Gastrointestinal disorders	Constipation	7	.	.	.
	Diarrhea	5	1	.	.
	Dry mouth	2	.	.	.
	Dyspepsia	.	1	.	.
	Flatulence	1	.	.	.
	Gastroesophageal reflux disease	2	2	.	.
	Gastrointestinal disorders - Other	2	.	.	.
	Gingival pain	1	.	.	.
	Hemorrhoids	1	.	.	.
	Mucositis oral	3	2	.	.
	Nausea	11	4	.	.
	Vomiting	2	.	.	.
General disorders and admin site conditions	Edema limbs	.	1	.	.
	Fatigue	16	5	.	.
	Fever	2	.	.	.
	Flu like symptoms	.	1	.	.
	Pain	3	1	.	.
Infections and infestations	Infections and infestations - Other	.	1	.	.
	Lung infection	.	.	1	.
	Papulopustular rash	1	.	.	.
	Sepsis	.	.	.	1
	Sinusitis	.	2	.	.
	Skin infection	.	1	.	.
	Upper respiratory infection	3	.	.	.
Investigations	Alanine aminotransferase increased	5	4	1	.
	Alkaline phosphatase increased	2	.	.	.
	Aspartate aminotransferase increased	7	2	1	.
	Neutrophil count decreased	.	1	3	2
	Weight loss	2	.	.	.
Metabolism and nutrition disorders	Anorexia	2	1	.	.
	Dehydration	.	3	.	.
	Hypomagnesemia	1	.	.	.
Musculoskeletal and connective tissue disorders	Arthralgia	3	2	.	.
	Bone pain	.	1	.	.
	Musculoskeletal and connective tissue disorder - Other, specify	1	.	.	.
	Myalgia	3	1	.	.
	Pain in extremity	.	1	.	.
Nervous system disorders	Dizziness	.	1	.	.
	Dysgeusia	3	.	.	.
	Headache	6	5	.	.
	Nervous system disorders - Other	3	.	.	.
	Peripheral sensory neuropathy	9	.	.	.
	Tremor	1	.	.	.
Psychiatric disorders	Anxiety	.	1	.	.
	Depression	.	2	.	.
	Insomnia	3	1	.	.
Renal and urinary disorders	Urinary incontinence	.	1	.	.
Reproductive system and breast disorders	Breast pain	1	1	.	.
Respiratory, thoracic and mediastinal disorders	Cough	.	1	.	.
	Dyspnea	1	3	.	.
	Hoarseness	1	.	.	.
	Pharyngolaryngeal pain	1	.	.	.
	Pneumonitis	.	.	1	.
	Postnasal drip	1	.	.	.
	Respiratory, thoracic and mediastinal disorders - Other	1	1	.	.
	Sore throat	2	.	.	.
Skin and subcutaneous tissue disorders	Alopecia	2	12	.	.
	Dry skin	3	.	.	.
	Hyperhidrosis	1	.	.	.
	Palmar-plantar erythrodysesthesia syndrome	1	.	.	.
	Periorbital edema	1	.	.	.
	Photosensitivity	1	.	.	.
	Rash acneiform	1	.	.	.
	Rash maculo-papular	2	2	.	.
	Skin/subcutaneous tissue disorders; Other, specify	1	.	.	.
Vascular disorders	Flushing	1	.	.	.
	Hot flashes	1	2	.	.
	Lymphedema	.	1	.	.
	Thromboembolic event	.	1	.	.

*Abbreviations: CTCAE *NCI Common Terminology Criteria for Adverse Events version 4.0

### MRI Imaging

**Fig. 1:**
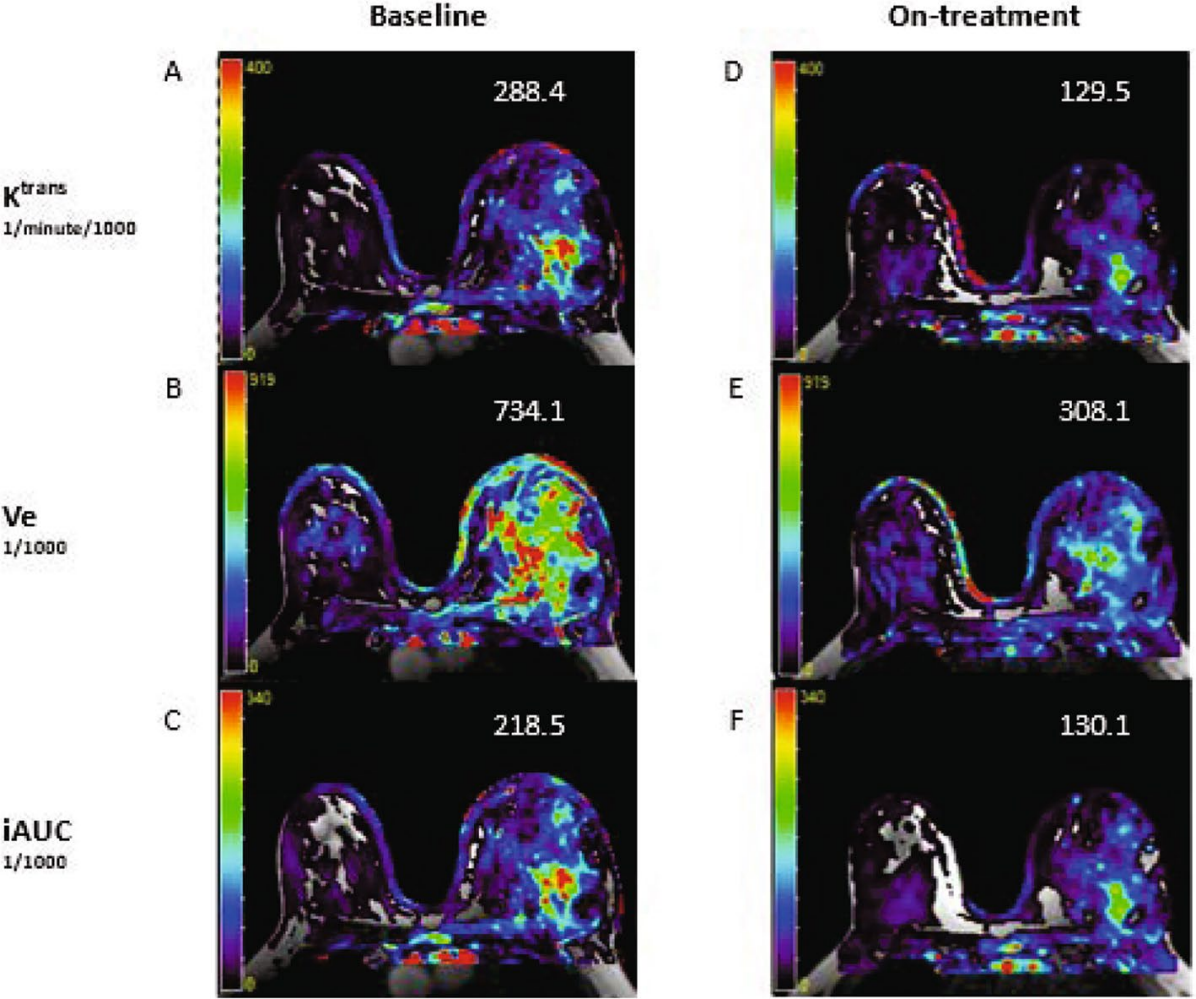
Example of DCE-MRI with decrease in K^trans^ (volume transfer constant from blood plasma to extravascular extracellular space), V_e_ (fractional volume of extravascular extracellular space), and iAUC (incremental area under the signal time intensity curve) on Day 8 on treatment compared to baseline. A 54-year-old woman with left inflammatory breast cancer (IBC) showed decrease in vascularity on treatment compared to baseline. A) Baseline K^trans^ B) Baseline V_e_ C) Baseline iAUC D) On-treatment K^trans^ E) On-treatment V_e_ F) On-treatment iAUC**.** DCE-MRI, dynamic contrast-enhanced magnetic resonance imaging

Ten patients (Cohort A (*n* = 5) and Cohort B (*n* = 5)) participated in the DCE-MRI sub-study. Nine had paired pre-treatment and on-treatment MRIs. Comparison of pre-treatment and on-treatment MRIs showed statistically significant decreases in tumor vascularization from baseline for K^trans^ (median 49.12), V_e_ (127.78), and iAUC (56.75) (*p*-values < 0.005). The magnitude of changes was not different when comparing Cohorts A and B. There was no significant change in K_ep_ or apparent diffusion coefficient (ADC) (Supplementary Table 3 in Additional File 1; Fig. 1).

### Transcriptomics

**Fig. 2:**
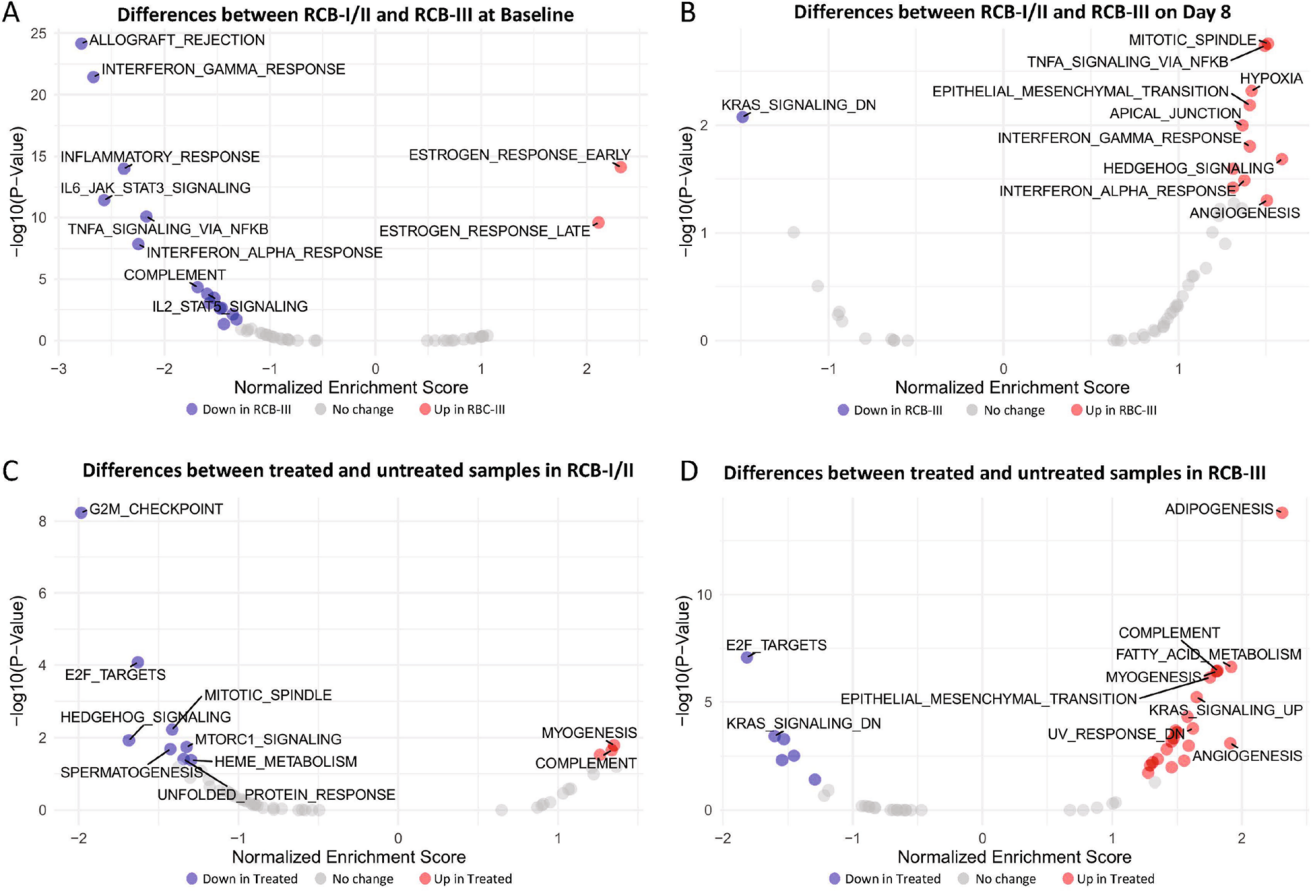
Volcano plots with gene enrichment analyses showing differences in gene expression between (**A**) tumors with RCB-I/II and RCB-III at baseline, (**B**) RCB-I/II and RCB-III at Day 8 of treatment, (**C**) pre- and on-treatment samples from RCB-I/II, and (**D**) pre- and on-treatment samples from RCB-III. RCB, residual cancer burden

Paired samples obtained at baseline and day 8 of treatment were collected in 21 (95%) patients and paired samples available for analysis in 15 (68%) patients. Comparison of gene expression at baseline between tumors in the RCB-I/II (*n* = 4) and RCB-III (*n* = 11) groups showed that those in the RCB-III group were more likely to have higher expression levels of estrogen signaling genes, and RCB-I/II were more likely to have higher expression levels of genes associated with inflammatory response and processes related to stress signaling (EMT, hypoxia, complement) (Fig. 2A; Supplementary Fig. 1 A in Additional File 1). In on-treatment samples collected at day 8, tumors in the RCB-III group showed overexpression of processes related to aggressive tumor biology (EMT, angiogenesis, hedgehog signaling) (Fig. 2B; Supplementary Fig. 1B in Additional File 1). In all patients, samples collected at baseline overexpressed many cell-proliferation-related hallmarks (Fig. 2C, D; Supplementary Fig. 1 C, D in Additional File 1) while in RCB-III, on-treatment samples overexpressed hallmarks reflective of aggressive cell biology (EMT, angiogenesis, TGF-β) as well as lipid metabolism (adipogenesis, fatty acid metabolism) (Fig. 2D; Supplementary Fig. 1D in Additional File 1).

### Tumor microenvironment (TME)

**Fig. 3:**
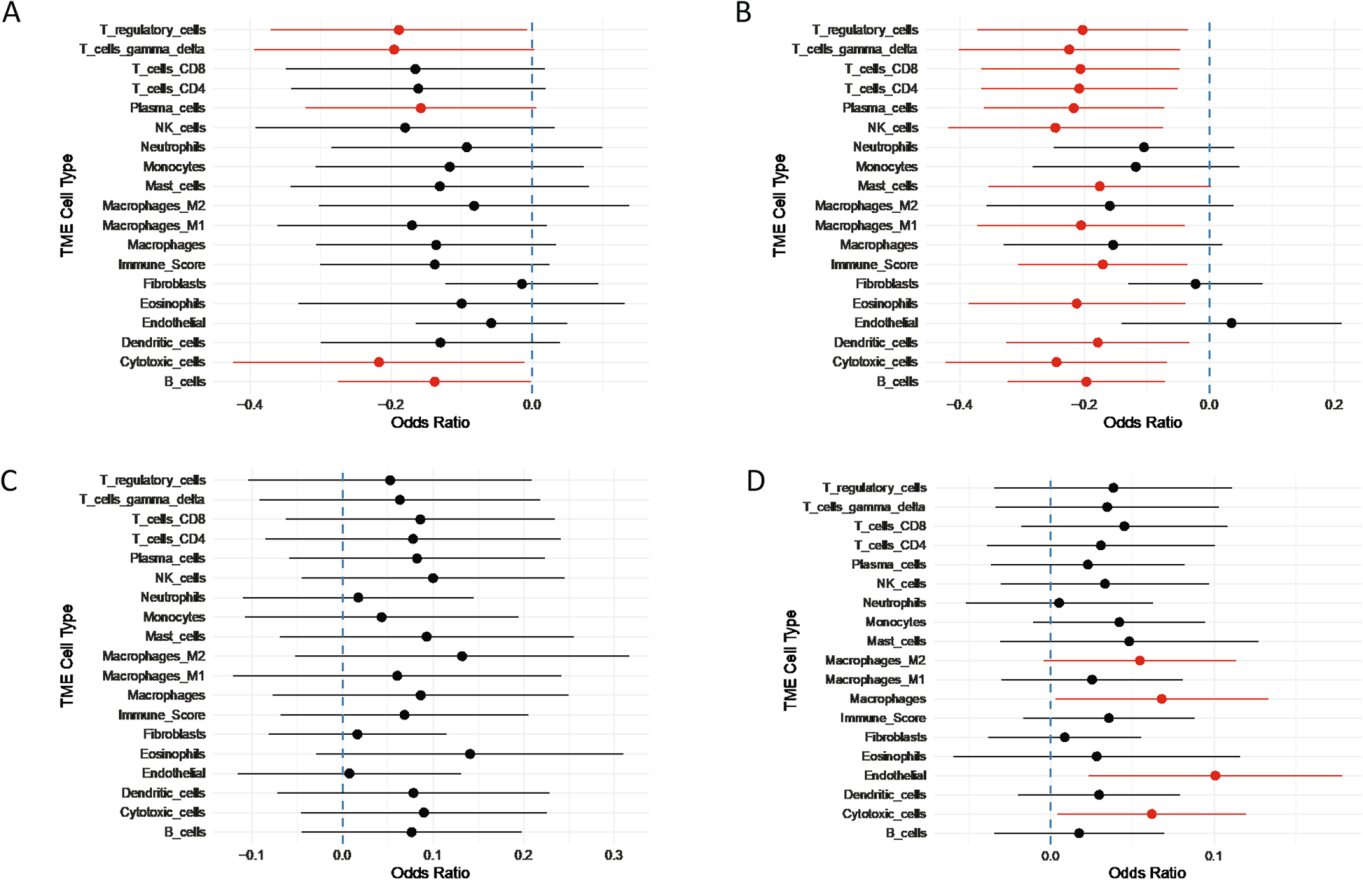
Forest plots showing difference in TME between (**A**) tumors with RCB-I or II (RCB-I/II) and RCB-III (RCB-III) at baseline, (**B**) RCB-I/II and RCB-III on Day 8 of treatment, (**C**) baseline and on-treatment samples from RCB-I/II, and (**D**) baseline and on-treatment samples from RCB-III. TME, tumor microenvironment; RCB, residual cancer burden

We observed higher levels of T regulatory cells (Tregs), gamma delta T cells, plasma cells, and B cells in the baseline TME of RCB-I/II compared to RCB-III (Fig. 3A). Comparing day 8 samples from RCB-I/II and RCB-III, RCB-I/II had more engagement of proinflammatory immune cells (e.g., mast cells, gamma delta T cells, M1 macrophages) (Fig. 3B). There was no difference in immune activation status between the pre- and on-treatment samples in RCB-I/II (Fig. 3C). In RCB-III, on-treatment samples had an increase in cells associated with immunosuppression (macrophages, particularly immunosuppressive macrophages called “M2” in the computational pipeline, and endothelial cells) as well as cytotoxic cells (Fig. 3D).

### Mutational analysis

Paired comparison of the mutational heterogeneity between baseline and on-treatment samples showed that in RCB-I/II, mutational heterogeneity decreased in 75% of cases and increased in 25% of cases (*n* = 4) (Supplementary Fig. 2 A in Additional File 1). In RCB III tumors, mutational heterogeneity decreased in 64% of cases, increased in 27% of cases, and remained stable in 1 case (9%) (*n* = 11). (Supplementary Fig. 2B in Additional File 1).

## Discussion

Among 22 patients, NAST with E-AC or AC-E resulted in a pCR rate of 5%, RCB-I rate of 9%, RCB-II rate of 32%, RCB-III rate of 50%. Despite the low pCR rate with E-AC or AC-E in this study, all patients were able to undergo MRM, 5-year EFS rate was 86%, and there were no unexpected safety signals. Decreases in tumor vascularization with treatment were detected by DCE-MRI parameters irrespective of initial chemotherapy received. Transcriptomic analysis revealed that patients with RCB-I/II were more likely to have higher expression levels of genes associated with inflammatory response and processes related to stress signaling at baseline, while on-treatment analysis for patients with RCB-III revealed overexpression of signatures related to cell proliferation and adipogenesis. Interestingly, after therapy RCB-III tumors had increased cytotoxic cells in addition to increased “M2” macrophages. While the M1/M2 dichotomy is an outdated definition of macrophage phenotype given the diversity of macrophage subsets identified in solid tumors, it suggests a suppressive phenotype of macrophages is activated in RCB-III tumors after therapy. It is possible that these suppressive macrophages inhibit the activity of the cytotoxic cells in the TME. Further work to determine a more precise phenotype of macrophages in these tumors along with their spatial location to cytotoxic cells is warranted.

Analysis of the TME from on-treatment biopsies showed increased engagement of proinflammatory cells such as mast cells and “M1” macrophages in RCB-I/II. In both RCB-I/II and RCB-III, mutational heterogeneity decreased after initiation of treatment.

While the pCR rate in this study was low, 5-year EFS was 86%, which is encouraging in this patient population with mostly ER + HER2-negative IBC. While pCR is frequently used as a primary endpoint in trials of neoadjuvant treatment, it is likely to be an imperfect surrogate for benefit. A prespecified exploratory analysis of EFS within RCB categories of the KEYNOTE-522 trial of neoadjuvant chemotherapy ± pembrolizumab in patients with early-stage, high-risk TNBC found that while EFS rates were improved with smaller amounts of residual disease regardless of treatment arm, the patients who derived the most benefit from pembrolizumab were those with RCB-II. Three-year EFS for patients with RCB-II was 76% if they were randomized to the pembrolizumab arm and 56% if they were randomized to no pembrolizumab (hazard ratio 0.52 [95% CI 0.32–0.82]). Outcomes by treatment in the other RCB categories did not show a significant difference. This finding suggests that while pCR is a helpful early endpoint, EFS or overall survival are likely more meaningful endpoints for evaluating the true impact of an intervention on patient outcomes [19].

This study only enrolled patients with IBC, a unique population with aggressive breast cancer, for whom current treatment recommendations are extrapolated from trials that primarily enrolled patients without IBC. CALGB 9741, the trial that established dose-dense AC-paclitaxel as standard of care chemotherapy in patients with node-positive breast cancer, did not include patients with T4 disease [20]. While the pCR rate of 5% (1/22) in this trial is low, given that 86% of patients had ER > 1% disease, it is not meaningfully different from previously reported pCR rates (6–14%) in institutional and national databases [13, 21–23]. Despite this low pCR rate, the freedom from recurrence after a median follow up of 76 months was much better than the previously reported 5-year RFS of 25% in patients predominantly with ER- IBC that did not have pCR after neoadjuvant chemotherapy [24]. Of note, these patients did not receive paclitaxel preoperatively. In a separate study, patients with HR +/HER2- IBC had 3-year RFS of 52% and 5-year RFS of 38% [13]. The findings of this study are consistent with recent data from the phase III JBCRG-M06/EMERALD trial that showed eribulin was non-inferior to taxane when combined with dual HER2 blockade for patients with advanced HER2-positive breast cancer [25]. Given the EFS rate in this study, investigation of this chemotherapy regimen in future trials may be merited.

The rate of micro-vessel density has been shown to be higher in archival IBC tissue compared to non-IBC tumors [26] and pro-angiogenic VEGF-family proteins are thought to play an important role in locoregional metastases to local lymph nodes in malignancy [27]. The decreased tumor vascularization post-NAST detected by DCE-MRI independent of treatment order in this study suggests a potential early signal of activity of these chemotherapeutic agents in this angiogenesis-dependent disease.

Transcriptomic analysis of pre- and on-treatment biopsies showed that RCB-III tumors were more likely to have higher expression levels of estrogen signaling genes and lower expression levels of inflammatory response genes at baseline, in keeping with findings from prior studies in IBC [28, 29]. Interestingly, treated samples from RCB-III were more likely to have increased expression of genes associated with adipogenesis and fatty acid metabolism. Prior studies have shown that increased adipogenesis is associated with unfavorable TME and shorter survival in patients with TNBC [30], and both adipocytes and fatty acid metabolism play important roles in cancer initiation, growth, metastasis, and response to treatment [31, 32]. It is possible that the macrophages found in these tumors may exhibit fatty acid metabolism, which is associated with poor clinical outcomes [33]. These findings in our study may suggest a role for lipid metabolism in acquiring resistance to chemotherapy in this population. This adipogenesis signal could also reflect the HR + status of the majority of the patients in this trial as fat is a source of estrogen production.

An earlier study of 23 patients with HER2-positive IBC who underwent baseline biopsy and day 8 on-treatment biopsy after loading doses of trastuzumab and pertuzumab found that the gene expression profiles most associated with having a pCR in this population were markers of adaptive immunity, innate immunity, and antigen presentation [34]. The difference in transcriptomic profile between this HER2-poistive IBC population and that seen in the population in this trial with patients with TNBC and majority HR + IBC may reflect a difference in gene expression resulting from treatment with HER2-targeting therapies only versus treatment with chemotherapy.

Analysis of the TME in on-treatment biopsies showed that samples from RCB-I/II were more likely to have increased proinflammatory immune cells, including mast cells and “M1” macrophages, compared to RCB-III. On-treatment biopsies from RCB-III had higher levels of cells associated with immunosuppression (including “M2” macrophages). A previous study of the TME in IBC showed that lower pretreatment mast cell density was significantly associated with achieving pCR and that mast cells in proximity to CD163 + monocytes/macrophages and tumor cells was associated with not achieving pCR [35]. Together, these findings suggest a potential mechanism of resistance and area of future investigation for therapeutic targets in IBC.

Overall, mutational heterogeneity decreased between baseline and on-treatment samples in both RCB-I/II and RCB-III, likely due to cancer cell death. Prior studies of the mutational landscape in IBC have not found major differences when comparing with non-IBC [36, 37]. Mutational heterogeneity remains a major hurdle in the treatment of breast cancer, limiting the utility of targeted agents and leading to resistance to therapy [38].

This study has several Limitations. The single arm design does not allow for direct comparison with a control treatment regimen to assess efficacy or impact on correlative investigations. Also, the small sample size Limits the conclusions that can be drawn from the data generated in this study, including the imaging findings. Thus, rather than definitive, the findings reported here should be considered hypothesis generating. Finally, we did not use IHC to confirm our transcriptomic findings. With acknowledgement of these limitations, this prospective, phase II trial enrolled patients with a rare form of breast cancer and provides important results describing this unique population who are often under-represented in clinical trials. This trial also reports the preplanned analysis of prospectively collected paired tumor samples obtained in 95% of patients and available for correlative analyses in 68% of patients.

In conclusion, despite the low pCR rate, all patients in this trial were able to undergo curative surgery. With a median follow-up of 76 months, 5-year EFS was 86% suggesting future exploration of eribulin or antibody–drug conjugate with eribulin payload may be justified in this population. Decreases in tumor vascularization with treatment were detected by DCE-MRI parameters irrespective of initial chemotherapy received, and transcriptomic and TME analyses highlighted adipogenesis/fatty acid metabolism and suppressive macrophages as potential mechanisms of resistance and targets for future investigation in this unique patient population.

## Supplementary Information

Additional file 1: Supplementary Figure 1. Volcano plots with genes showing differences in gene expression. Supplementary Figure 2. Paired comparison of the number of mutations between baseline and treated samples from tumors with RCB-I or II (RCB-I/II) (A) and RCB-III (B). Supplementary Table 1. Representativeness of Study Participants. Supplementary Table 2. Clinical and imaging response. Supplementary Table 3. DCE-MRI Tumor Vascularization

## Data Availability

Data are available upon request from the corresponding author, Filipa Lynce, MD.

## Abbreviations

ADC: Apparent diffusion coefficient
AE: Adverse event
AIF: Arterial input function
ASCO/CAP: American Society of Clinical Oncology/College of American Pathologists
CA: Contrast agent
CI: Confidence interval
CR: Complete response
CTCAE: NCI Common Terminology Criteria for Adverse Events
DCE-MRI: Dynamic contrast-enhanced magnetic resonance imaging
ddAC: Dose dense doxorubicin (Adriamycin) and cyclophosphamide (Cytoxan)
DF/HCC: Dana-Farber/Harvard Cancer Center
DSMC: Data safety monitoring committee
DWI: Diffusion-weighted imaging
E: Eribulin
ECOG: Eastern Cooperative Oncology Group
EES: Extravascular extracellular space
EFS: Event-free survival
EMT: Epithelial-mesenchymal transition
ER +: Estrogen receptor-positive
ET: Endocrine therapy
FOV: Field of view
FSPGR: Fast spoiled gradient recalled echo
GSEA: Gene set enrichment analysis
HER2-: Human epidermal growth factor receptor 2-negative
HIV: Human immunodeficiency virus
iAUC: Incremental area under the signal time intensity curve
IBC: Inflammatory Breast Cancer
IDC: Invasive ductal carcinoma
IHC: Immunohistochemistry
ILC: Invasive lobular carcinoma
ISH: In situ hybridization
Ktrans: Volume transfer constant from blood plasma to extravascular extracellular space
MRM: Modified radical mastectomy
NAST: Neoadjuvant systemic therapy
OCT: Optimal cutting temperature
OS: Overall survival
pCR: Pathologic complete response
PK: Pharmacokinetic
PMRT: Postmastectomy radiation therapy
PR: Partial response
RCB: Residual cancer burden
RT: Reverse transcription enzyme
RT-qPCR: RT-quantitative polymerase chain reaction
SD: Stable disease
SPAIR: Spectral Adiabatic Inversion Recovery
TME: Tumor microenvironment
TMM: Trimmed Mean of M-values method
TPC: Treatment of physician’s choice
Tregs: T regulatory cells
Ve: Fractional volume of extravascular extracellular space
VST: Variance stabilizing transformation
