# Supplementary material for: Neoadjuvant therapy with eribulin, doxorubicin and cyclophosphamide for patients with HER2-negative inflammatory breast cancer: a phase II study
Source: Breast Cancer Res. 2025 Sep 29;27:171. doi: 10.1186/s13058-025-02108-4 (PMC12482238; doi:10.1186/s13058-025-02108-4)
Supplement: Supplementary file 1 — Additional file 1: Supplementary Figure 1. Volcano plots with genes showing differences in gene expression. Supplementary Figure 2. Paired comparison of the number of mutations between baseline and treated samples from tumors with RCB-I or II (RCB-I/II) (A) and RCB-III (B). Supplementary Table 1. Representativeness of Study Participants. Supplementary Table 2. Clinical and imaging response. Supplementary Table 3. DCE-MRI Tumor Vascularization [file 13058_2025_2108_MOESM1_ESM.pdf]

Supplement

Neoadjuvant Therapy with Eribulin, Doxorubicin and Cyclophosphamide for Patients with HER2-Negative Inflammatory Breast Cancer: A Phase II Study

Supplementary Figure 1: Volcano plots with genes showing differences in gene expression.................... 2

Supplementary Figure 2: Paired comparison of the number of mutations between baseline and treated samples from tumors with RCB-I or II (RCB-I/II) (A) and RCB-III (B)..3

Supplementary Table 1: Representativeness of Study Participants..4

Supplementary Table 2: Clinical and imaging response... 5

Supplementary Table 3: DCE-MRI Tumor Vascularization ……………………………………………...6

Supplementary Figure 1: Volcano plots with genes showing differences in gene expression between (A) patients with RCB-I/II and RCB-III at baseline, (B) RCB-I/II and RCB-III at Day 8 of treatment, (C) pre- and on-treatment samples from RCB-I/II, and (D) pre- and on-treatment samples from RCB-III.

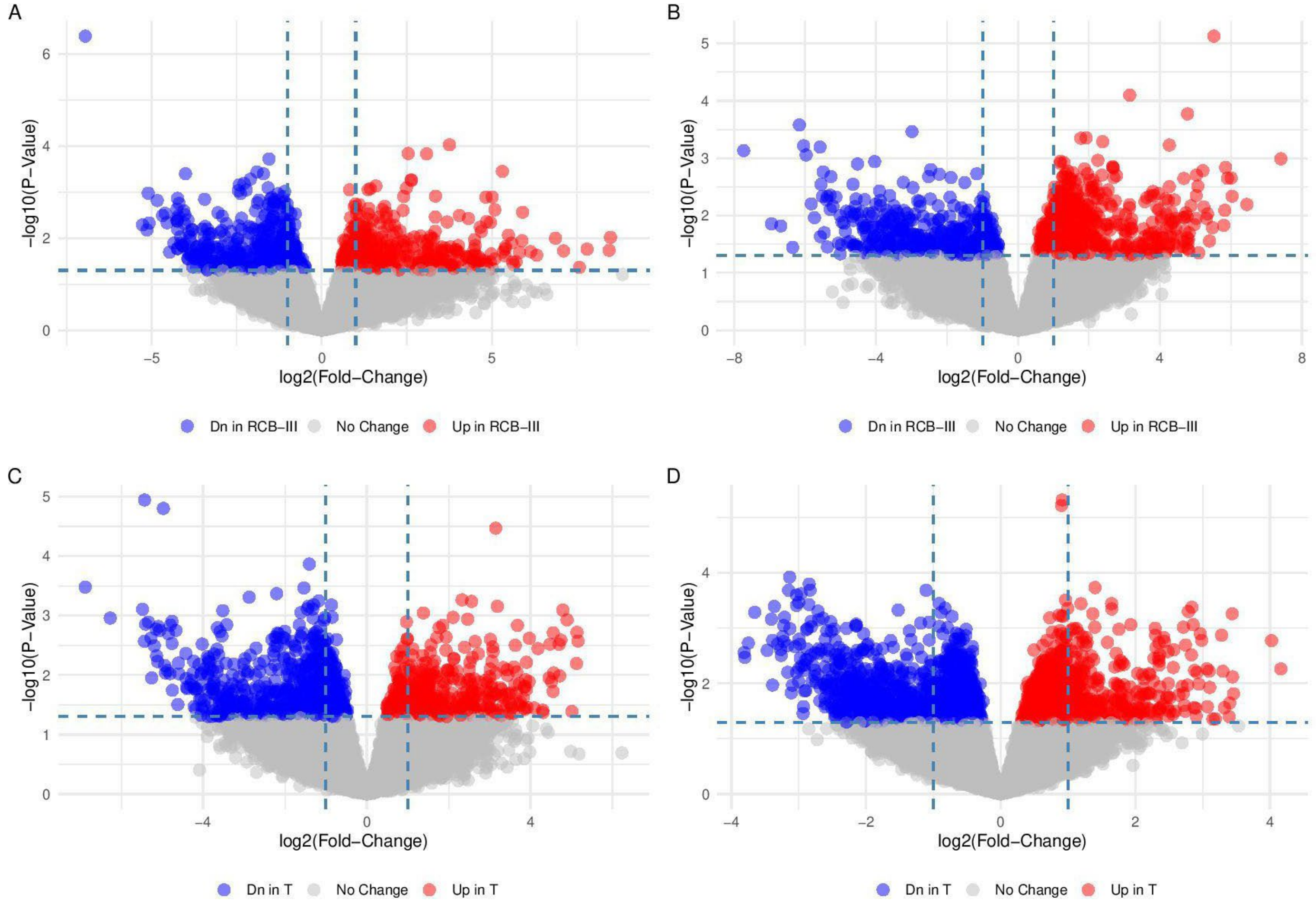

Supplementary Figure 2. Paired comparison of the number of mutations between baseline and treated samples from tumors with RCB-I or II (RCB-I/II) (A) and RCB-III (B).

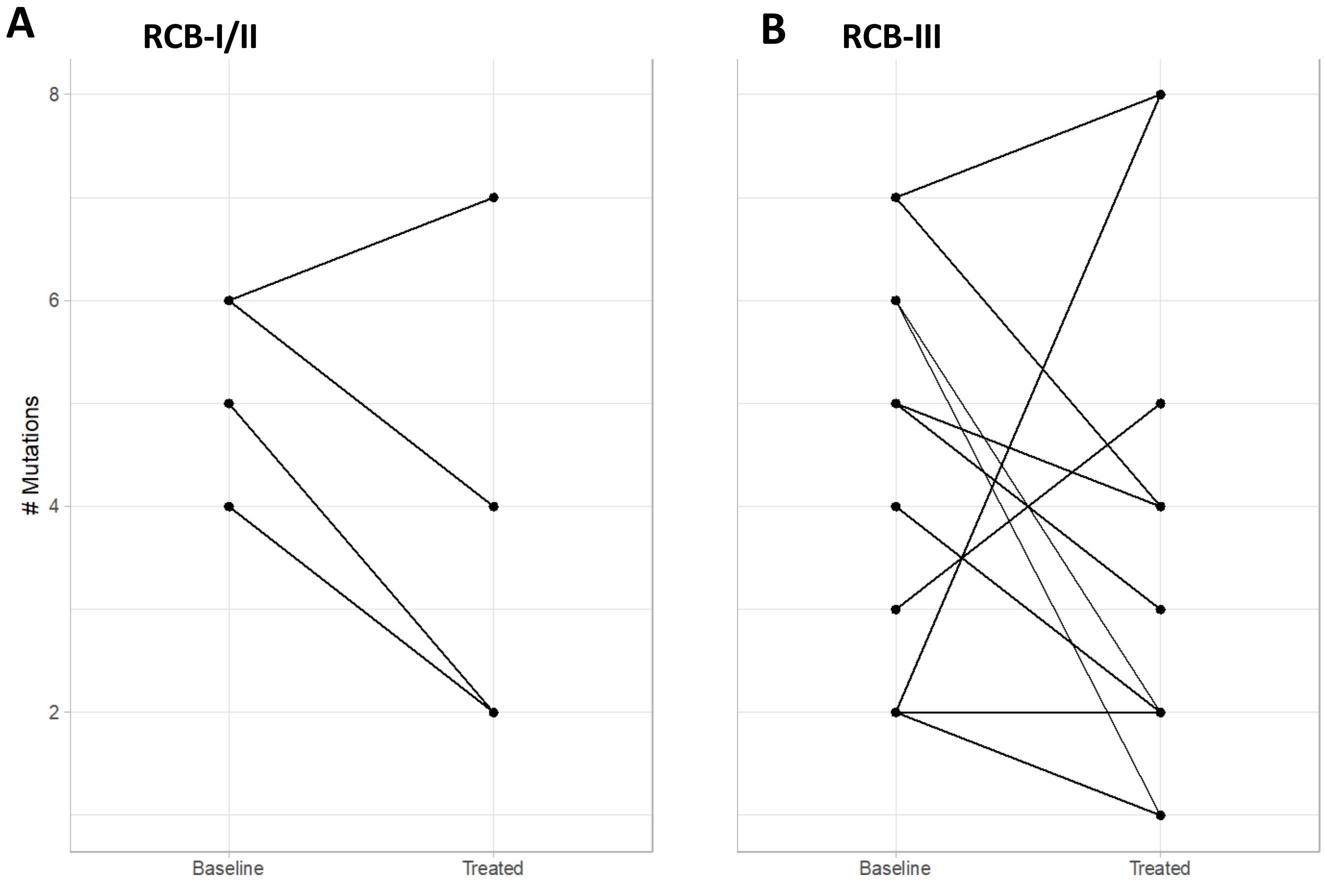

**Supplementary Table 1:** Representativeness of Study Participants	
Cancer type(s)/ subtype(s)/ stage(s)/condition	HER2-Negative Inflammatory Breast Cancer (IBC)
Considerations related to:	
Sex	IBC, as all other subtypes of breast cancer, is a predominantly female disease and is rare in men. Male breast cancer represents only between 0.5 and 1% of all breast cancers diagnosed each year. IBC incidence is around 2- 3% among all breast cancer subtypes in the overall largely female population, but its incidence in males is lower when compared to the other subtypes.
Age	The median age at the time of all breast cancer diagnoses is around 63, while in IBC it is closer to 57.
Race/ethnicity	The overall incidence of IBC from 1973 to 2015 is 2.76 (2.73, 2.79) cases per 100,000 people, with White patients having an incidence rate of 2.63 (2.60, 2.67), Black patients 4.52 (4.39, 4.65), and patients of other race 1.84 (1.76, 1.93). The overall IBC relative 5-year survival rate is 40.5% (39.0%, 42.0%), 42.5% (40.7%, 44.3%), and 29.9% (26.6%, 33.3%) for White patients and Black patients, respectively.
Geography	IBC rates vary geographically in the United States and are influenced by social and economic factors. Counties with above- average IBC rates tend to be clustered together and are significantly different from low-rate counties in terms of race, poverty, and urbanicity.
Other considerations	
Overall representativeness of this study	The age distribution of our study is similar to the average age distribution of IBC in the literature, with a median age of 58.<br><br>The majority of patients in our study were White (95.4%). Although this doesn't reflect the distribution of IBC in the population, this disparity is explained by the fact that our study population was limited to our institution's main site in Boston.<br><br>As breast cancer in rare in men, no male IBC patients were recruited, although this was not an exclusion criterium.

Supplementary Table 2: Clinical and imaging response

		Treatment Cohort				Overall	
		Eribulin>ddAC		ddAC>Eribulin			
			%		%		%
N enrolled		16	100.0	6	100.0	22	100.0
Progression on protocol treatment?							
No		16	100.0	6	100.0	22	100.0
Best clinical response by exam							
cCR		7	43.8	3	50.0	10	45.5
cPR		8	50.0	3	50.0	11	50.0
SD		1	6.3	.	.	1	4.5
Imaging response pre-surgery							
Partial Response		14	87.5	5	83.3	19	86.4
Stable Disease		2	12.5	1	16.7	3	13.6
Best clinical response by exam	Imaging response pre-surgery						
cCR	Partial	6	37.5	3	50.0	9	40.9
	Stable Disease	1	6.3	.	.	1	4.5
cPR	Partial	8	50.0	2	33.3	10	45.5
	Stable Disease	.	.	1	16.7	1	4.5
SD	Stable Disease	1	6.3	.	.	1	4.5

Abbreviations: **ddAC,** dose-dense doxorubicin (Adriamycin) and cyclophosphamide (Cytoxan) ; **cCr,** complete clinical response; cPR, clinical partial response; **SD,** stable disease

Supplementary Table 3: DCE-MRI Tumor Vascularization

Measurement	Median difference pre and on-treatment (25th, 75th percentile)	p-value	n pairs	Median pre-post in A (25th, 75th)	Median pre-post in B (25th, 75th)	p-value	n for A	n for B
K^{trans}	49.1 (34.8, 110.2)	0.008	9	42.6 (26.8, 68.8)	63.4 (34.8, 110.2)	0.73	4	5
V_E	127.8 (82.7, 197.7)	0.004	9	88.8 (77.6, 113.4)	197.7 (127.8, 215.3)	0.29	4	5
K_{EP}	17.8 (0.5, 203.1)	0.13	9	-0.9 (-45.0, 59.2)	57.2 (17.8, 225.5)	0.19	4	5
iAUC 90	56.7 (51.1, 102.9)	0.004	9	58.9 (45.3, 75.7)	51.9 (51.1, 102.9)	1.00	4	5
ADC	-43.1 (-82.9, -12.1)	0.07	9	-56.6 (-78.6, -41.6)	-12.2 (-82.9, 29.0)	0.56	4	5

Abbreviations: **DCE-MRI,** dynamic contrast enhanced magnetic resonance imaging; **ADC,** apparent diffusion coefficient; K^{trans}, volume transfer constant from blood plasma to extravascular extracellular space; V_e, fractional volume of extravascular extracellular space; K_{ep}, rate constant between extravascular extracellular space and blood plasma; iAUC, initial area under the signal time intensity curve
